# A review of through-knee amputation

**DOI:** 10.1177/17085381211045183

**Published:** 2021-11-29

**Authors:** Brieuc Panhelleux, Joseph Shalhoub, Anne K Silverman, Alison H McGregor

**Affiliations:** 1 Department of Surgery and Cancer, 4615 Imperial College London, London, UK; 2 Imperial Vascular Unit, 4615 Imperial College Healthcare NHS Trust, London, UK; 3 Department of Mechanical Engineering, 3557 Colorado School of Mines Golden, CO, USA

**Keywords:** Amputee, Gritti-Stokes, Mazet, knee disarticulation, surgical technique

## Abstract

**Objectives:**

Through-knee amputation is an umbrella term for several different surgical techniques, which may affect clinical and functional outcomes. This makes it hard to evaluate the benefits and need for a through-knee amputation approach. This article seeks to (1) determine the number of through-knee amputation performed compared with other major lower limb amputations in England over the past decade; (2) identify the theoretical concepts behind through-knee amputation surgical approaches and their potential effect on functional and clinical outcomes and (3) provide a platform for discussion and research on through-knee amputation and surgical outcomes.

**Methods:**

National Health Service Hospital Episodes Statistics were used to obtain recent numbers of major lower limb amputations in England. EMBASE and MEDLINE were searched using a systematic approach with predefined criteria for relevant literature on through-knee amputation surgery.

**Results:**

In the past decade, 4.6% of major lower limb amputations in England were through-knee amputations. Twenty-six articles presenting through-knee amputation surgical techniques met our criteria. These articles detailed three through-knee amputation surgical techniques: the classical approach, which keeps the femur intact and retains the patella; the Mazet technique, which shaves the femoral condyles into a box shape and the Gritti-Stokes technique, which divides the femur proximal to the level of the condyles and attaches the patella at the distal cut femur.

**Conclusions:**

Through-knee amputation has persisted as a surgical approach over the past decade, with three core approaches identified. Studies reporting clinical, functional and biomechanical outcomes of through-knee amputation frequently fail to distinguish between the three distinct and differing approaches, making direct comparisons difficult. Future studies that compare through-knee amputation approaches to one another and to other amputation levels are needed.

## Introduction

Major lower limb amputations (MLLAs) are common in dysvascular and trauma populations, with increases in recent years attributed in part to recent military conflicts.^1^ Furthermore, there has been an increase in MLLA in dysvascular patients, representing 80% of all MLLA, owing to the rise in vascular pathologies and diabetes worldwide.^2^ While the total number of major lower limb amputations has increased, through-knee amputations (TKAs) represent only a small proportion. In 2004, TKA was performed in less than 2% of all amputations,^3^ but the current incidence of TKA is unclear. Historically, TKA was associated with a number of reported postoperative complications and failures linked to blood supply and viability of tissues,^4^ resulting in above-knee amputations (AKAs) being preferred to TKA by many surgeons.^5^ Moreover, surgical techniques for TKA vary and the relative benefits of individual techniques are poorly understood.

There are limited outcome data relating to TKA which, coupled with the absence of a standardised surgical technique, makes comparison of TKA to AKA both a challenge and a dilemma. While some studies seek to compare outcomes of TKA to AKA, they often fail to specify the surgical technique(s) performed, preventing objective comparisons. There is a need to determine if TKA is a viable surgical technique and when it should be used. To inform such surgical guidelines, the influence of surgical technique on functional and clinical outcomes must be determined, instead of basing decisions on theoretical advantages and disadvantages alone.

### Number of through-knee amputations procedures in the past decade

The National Health Service (NHS) in England annually reports data relating to all hospital admissions, including procedures performed, which are made publicly available in the Hospital Episodes Statistics (HES) database.^6^ The HES database was searched to obtain the number of major lower limb amputation procedures performed each year in England from April 2011 to March 2020, according to the International Classification of Diseases 10^th^ revision (ICD10) codes. We compared four categories: amputation at or proximal to the hip (X09.1 and X09.2), amputation of leg above knee (X09.3), amputation of leg through knee (X09.4) and amputation of leg below knee (X09.5). Before 2011, lower limb amputations were reported with less precision, and 2020 was the last published data available as of May 2021.

A consistent number of major lower limb amputations, including amputation through the knee, were performed over the past decade in England (Table 1). An average of 242 TKA procedures were performed each year, representing on average 4.6% of all major lower limb amputations. Clearly TKA is still performed but it is not clear why this technique was performed less compared to other approaches or what specific TKA surgical technique was used. The data indicate that people having a TKA procedure have a mean age of 64 years, whereas those having an AKA had a mean age of 70 years. People receiving an amputation at a younger age will live longer with their amputation and therefore require particular attention to the effects of amputation on functional and biomechanical outcomes, as well as long-term health implications.^7,8^ However, survival rate is dependent on multiple factors including aetiology and co-morbidities. It is therefore critical that patients receive the amputation that will best serve them and result in better physical, clinical and functional outcomes.^9^

**Table 1.** Yearly average numbers of major lower limb amputation (MLLA) procedures performed in England from April 2011 to March 2020 and as a percentage of total MLLA procedures between 2011 and 2020.

Procedure	Yearly average 2011–2020 (±standard deviation)	% of total MLLA procedures
Amputation at or proximal to the hip (X09.1 and X09.2)	76 (±9)	1.5
Amputation of leg above knee (X09.3)	2457 (±97)	47.4
Amputation of leg through knee (X09.4)	242 (±24)	4.6
Amputation of leg below knee (X09.5)	2413 (±86)	46.5

### Objectives

TKA prevails as a surgical option; thus, this article seeks to identify the theoretical concepts behind the different surgical approaches to TKA and their potential effect on functional and clinical outcomes. A 2016 literature review of the outcomes of dysvascular TKA and AKA patients identified a small number of articles presenting outcomes of TKA compared to outcomes of AKA^5^ but there were insufficient articles to perform a comparison of TKA to AKA. Moreover, the different surgical techniques for TKA were not documented, restricting meaningful comparisons between TKA techniques, and between TKA and AKA approaches.

While there are insufficient objective data to directly compare biomechanical and functional outcomes between techniques, this review aims to highlight the outcomes reported. We thus highlight the need for more objective and clear comparisons of the different TKA surgical techniques and of TKA to AKA to better define the place of TKA in LLA. This review will explore the need for a study to compare the outcomes of different TKA surgical techniques and AKA with a view to developing future surgical guidelines.

## Surgical techniques for through-knee amputations

The literature available on the surgical techniques of TKA does not provide the ability to do a meta-analysis. However, we systematically searched the literature for articles describing surgical techniques.

### Eligibility criteria

Articles describing a surgical technique, or the modification of a surgical technique, for TKA were included in this review. Case studies and cohort studies relating to adults with lower limb amputations of varying aetiologies were included; however, the three studies found on paediatric amputations were of a single case amputation for very rare congenital diseases and none offered novel surgical approaches, rather focusing on the congenital disease. Articles available in either the English or French language were included and between 1946 and June 2021; however, no articles meeting the inclusion criteria were published prior to 1966.

### Systematic search

The MEDLINE (since 1946) and EMBASE (since 1947) databases were systematically searched; the search strategy included a combination of synonyms of through-knee (OR TKA, Mazet, Gritti-Stokes) AND amputation (OR exarticulation, disarticulation) AND surgery (OR operation, surgical technique) (exemplar search strategy in Appendix 1). Additional articles were identified by reviewing reference lists of the included articles, prior to full-text screening.

### Study selection

Duplicates were removed from the identified articles and one reviewer (BP) reviewed all titles and excluded articles not related to lower-limb amputations. Two independent reviewers (BP and AM) assessed the remaining abstracts and subsequently the full-text articles using the systematic review software Covidence (Veritas Health Innovation, Melbourne, Australia, www.covidence.org) for inclusion or exclusion according to a priori criteria. Any discrepancies were discussed, with any disagreement resolved through a third author (AS). The articles were then read for extraction of descriptions of the surgical techniques, including treatment of the patella and femoral condyles, tendon attachment, flaps, as well as the participant sample, aetiology and study outcomes.

### Study selection

The search identified 11,832 articles, of which 6195 articles were removed as duplicates. The remaining 5637 records were screened from, producing a list of 209 full-text articles for inclusion. The references of these articles were reviewed revealing a further 12 articles for inclusion, which resulted in 221 articles progressing to full-text assessment (Appendix 2). Upon reading the full texts, 195 articles did not meet the inclusion criteria, resulting in 26 articles being included in the review (Table 2).

**Table 2.** Summary of included articles with surgical techniques, presented patients and reported outcomes.

Author (date)	Surgical technique	Keeps patella	Re-attaches patella or quadriceps tendon to cruciate ligaments	Shaves condyles	Horizontal flap	Sagittal flap	Number of participants (aetiology)	Outcomes measured
Newcombe (1972)^18^	Classical	**✓**			**✓**		41 (dysvascular)	Survival rate, incidence of healing and time from amputation to fitting
Baumgartner (1979, 1983, 2011)^4,11,13^	Classical	**✓**	**✓**			**✓**	None presented	None presented
Vaucher (1982)^21^	Classical	**✓**	**✓**	**✓**		**✓**	71 (mixed)	Survival rate
Jensen (1982)^20^	Classical	**✓**	**✓**			**✓**	71 (dysvascular)	Postoperative course, prosthetic fitting
Vitali (1987)^19^	Classical	**✓**	**✓**		**✓**	**✓**	None presented	None presented
Albers (1994)^16^	Classical	**✓**			**✓**		6 (cancer)	Residual limb healing
Bowker (2000)^14^	Classical	**✓**	**✓**		**✓**		77 (mixed)	Survival and healing rates
Anderson (2005)^17^	Classical	**✓**	**✓**		**✓**		None presented	None presented
Polfer (2019)^15^	Classical	**✓**	**✓**		**✓**		10 (trauma)	Functional outcomes surveys
Eid-Arimoku (2019)^12^	Classical	**✓**	**✓**			**✓**	None presented	None presented
Mazet (1966)^23^	Mazet			**✓**	**✓**		8 (mixed)	Ability to walk with prosthesis
Weale (1969)^25^	Mazet			**✓**	**✓**		21 (unspecified)	Postoperative state, healing rate and maintenance of ambulation
Burgess (1977)^10^	Mazet		**✓**	**✓**	**✓**	**✓**	None presented	None presented
Bowker (2000)^14^	Mazet		**✓**	**✓**	**✓**		77 (mixed)	Survival and healing rates
Cull (2001)^22^	Mazet		**✓**	**✓**	**✓**		12 (dysvascular)	Survival and healing rates and maintenance of ambulation
Morse (2008)^24^	Mazet			**✓**	**✓**		50 (dysvascular)	Survival, maintenance of ambulation and independent living status
Shackleton (1966)^28^	Gritti-stokes	**✓**		**✓**	**✓**		13 (unspecified)	Re-amputation and healing rates
Martin (1967)^26^	Gritti-stokes	**✓**		**✓**	**✓**		237 (dysvascular)	Survival, healing, re-amputation and maintenance of ambulation
Doran (1978)^31^	Gritti-stokes	**✓**		**✓**	**✓**		134 (dysvascular)	Survival, healing rate, maintenance of ambulation
Beacock (1983)^30^	Gritti-stokes	**✓**		**✓**	**✓**		247 (dysvascular)	Survival, healing rate and prosthetic use
Vitali (1986)^19^	Gritti-stokes	**✓**		**✓**	**✓**		None presented	None presented
Nellis (2002)^27^	Gritti-stokes	**✓**		**✓**	**✓**		63 (unspecified)	Survival and healing rate
Taylor (2012)^9^	Gritti-stokes	**✓**		**✓**	**✓**		None presented	None presented
Albino (2014)^29^	Gritti-stokes	**✓**	**✓**	**✓**	**✓**		46 (unspecified)	Healing and complications and maintenance of ambulation
Joseph (2017)^32^	Gritti-stokes	**✓**		**✓**	**✓**		9 (unspecified)	Intraoperative and postoperative outcomes
Theriot (2019)^33^	Gritti-stokes	**✓**		**✓**	**✓**		16 (dysvascular)	Estimated blood loss, operative time and perioperative and postoperative complications

Articles have been consistently published each decade since 1960, with a peak in 1980–1989 (Appendix 3), reflecting continued interest in TKA.

### Techniques

**Figure 1.**
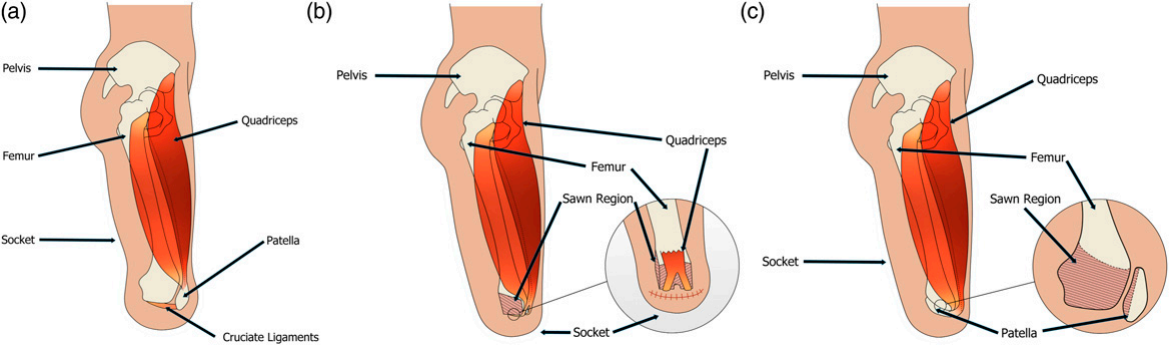
Schematic of the residual limb after through-knee amputation following different techniques. (a) Classical approach: the patella is preserved, and the patellar tendon is attached to the cruciate ligaments. (b) Mazet technique: the femoral condyles are shaved, the patella is removed and the quadriceps tendon is attached to the cruciate ligaments. (c) Gritti-Stokes technique: the femur is divided transversally, and the patella is attached at the distal cut end of the femur.

All TKA surgical techniques have some procedures and recommendations in common. In the procedure, the knee joint capsule is opened, and the knee is flexed for better access to the ligaments and posterior muscles. The popliteal artery and vein are carefully ligated, preserving the superior geniculate arteries, and the tibial and common peroneal nerves are transected under tension. Avoiding skin tension after flap closure is critical for successful residual limb healing.^10^ However, different approaches have been reported regarding the femoral condyles and the patella (Figure 1).

#### Classical approach

In the classical approach [Figure 1(a)], the femur is left intact,^4,11–20^ except for the technical modification described by Vaucher and Blanc^21^ where the femoral condylar cartilage and corners are excised to reduce the size of the condyles. The menisci are removed, except by Baumgartner (1979) who reported that the menisci could ‘add cover’ to the weight bearing femoral condyles in non-vascular patients.^13^ The patella is retained, and the patellar tendon is attached to the cruciate ligaments; however some authors do not detail if or where the patella tendon is re-attached.^16^

Flaps can be sagittal, lying posteriorly, vertically between the condyles^12,13,19–21^ or transverse, with the scar horizontally at the distal end of the residual limb, below, anteriorly or posteriorly.^14–18^ A preferred technique regarding flaps has not emerged. A dorsal flap uses the gastrocnemius to cover the femoral condyles, with the scar lying anteriorly and horizontally away from the weight-bearing area.^14–16^ A ventral flap, with the scar lying posteriorly and horizontally, allows thicker skin to be used for coverage.^10^ ‘Fish mouth’ flaps of equal lengths can be used with the scar lying on the most distal aspect of the residual limb.^18^

#### Mazet technique

In the Mazet technique [Figure 1(b)], the femoral condyles are shaved laterally and distally to obtain a ‘box’ shape, keeping the adductor magnus insertion to preserve muscle function.^10,14,22–24^ The patella is removed, and the quadriceps tendon is attached to the cruciate ligaments or the posterior flap tissue.^19^ This technique results in a conical shaped residual limb.^10,22,24^ The box-shaped femoral condyles provide a broad weight-bearing surface while reducing the bulge of the condyles.^23^ Weale (1966) followed a similar approach but cut the femur at the junction between the shaft and condyles, more proximal than the other reports presenting a version of the Mazet technique.^25^

The flaps in the Mazet technique are like those in the classical approach but result in reduced bulge due to the shaving of the femoral condyles. The removal of the condyles allows for more tissue and less tension at the distal end of the residual limb which may improve surgical outcomes.

#### Gritti-Stokes

The Gritti-Stokes technique is sometimes considered as an above-knee amputation because the amputation is supracondylar [Figure 1(c)]. This technique, however, allows for distal weight bearing on the femur, hence its inclusion as a through-knee amputation. The femur is cut transversally at either 0°,^26–29^ 10–15°^9,19^ or 45° to the horizontal.^30–33^ The posterior surface of the patella is shaved down to the cancellous bone and attached, with tension, to the distal cut end of the femur for weight bearing. The patellar tendon is attached to the posterior flap to prevent movement of the patella and potential damage to the soft tissue.^9,26–33^

A longer anterior flap for a posterior scar is performed according to the majority of descriptions.^9,26–28,30–33^ However, Albino et al. proposed a gastrocnemius flap, perhaps as they report division of the femur more distally than the other descriptions.^29^

### Comparison of techniques

TKA has different technical surgical approaches, with some having further surgical modifications that could affect functional and clinical outcomes. The classical TKA approach offers a broader weight-bearing surface by keeping the femoral condyles intact. Surgeons and prosthetists postulate that keeping the patella and attaching the patellar tendon to the cruciate ligaments affords additional stabilisation of the quadriceps muscles and improved proprioception,^14,19^ but this has not been verified. In contrast, the Mazet approach provides a flat surface for an even load distribution at the distal end of the femur, and the shorter thigh length allows the prosthetic knee to align with the contralateral knee and provides good muscle control and proprioception as the muscles are left intact.^19^ The shorter and less bulbous residual limb created with the Mazet technique improves cosmesis of the socket and prosthesis, with some stating it facilitates fitting of suction sockets.^10,22,24,25^ However, this technique requires shaving of the femoral condyles, which insults the bone potentially complicating healing, especially in those with dysvascular disease. Finally, the Gritti-Stokes technique is similar to AKA as the femoral division is supracondylar; however, weight-bearing remains distal as the patella is attached to the cut end of the femur, which is alleged to help with muscle control and proprioception.^26,33^ This technique also provides a shorter residual limb. However, this technique can fail at the femora-patellar union, with the potential for non-union, mal-union, bone necrosis and associated additional complications.^20,25^ For Gritti-Stokes amputated patients, the line of weight transmission lies behind the residual limb and with the potential to lead to greater mechanical stress on the thigh than the other techniques.^19^

In TKA, a dorsal flap using the gastrocnemius to cover the distal part of the femur allows for a large muscle belly, offering good coverage. The longer flap improves healing, with patients more likely to walk with a prosthesis and consequently derive improved functional outcomes.^14–16^ A ventral flap uses the skin from the front of the knee which can adapt to weight-bearing,^10^ but it is often associated with delayed wound healing and reduced soft tissue for bone coverage.^16^

In the literature, articles report the same surgical technique but fail to reference its origins or their adaptations. This has led to heterogeneity in surgical approaches with no clear consensus on approach. This variety of surgical approaches combined with different populations groups (e.g. age, co-morbidities, mobility and reason for amputation) further complicated studies comparing outcomes.^29^

The choice of surgical technique and flap configuration depends to a variable extent on the patient’s condition and presentation and the availability of healthy tissue in the context of significant tissue loss in severe arterial disease or trauma.^14,16,29^ The goals of treatment also vary and influence the decision to proceed with TKA. If the aim of amputation is for the patient to regain mobility using a prosthetic limb or manual wheelchair, then TKA is a viable option to facilitate prosthetic fitting, transfers and seated balance. However, if the patient is unlikely to mobilise and has poor tissue health, then the operating surgeon may prefer an AKA thereby limiting complications.

### Outcomes reported

Murakami (2016) reviewed outcomes of TKA in dysvascular patients noting no differences in residual limb health, prosthetic ambulation and mobility, functional outcomes with tests and questionnaires such as the sickness impact profile (perceived health status) between TKA surgical techniques and between TKA and AKA.^5,34^

Of the 26 articles presenting TKA surgical techniques, 8 present both operative and functional outcomes,^20,22,24–26,29–31^ eight present only operative outcomes,^14,16,18,21,27,28,32,33^ two present only functional outcomes^15,23^ and eight present no outcomes (Table 2).^4,9–13,17,19^

Survival rates between techniques are inconsistent, ranging from 84% to 100% for all techniques.^12,16,19,23,24,29^ For the classical approach, survival rates of 56% after one year^18^ and 72% after an undefined period^21^ are reported. For the Mazet technique, 60% at three years and 44% at five years are reported.^24^ The Gritti-Stokes technique reports survival rates of 63% after one year^29^ and 22% after five years.^30^ However, survival outcomes depend more on patient-related factors than technical surgical aspects^35^ and are thus a poor comparator.

Primary healing rates have been reported between 60 and 80%, delayed healing rates around 20% and failure to heal for the remaining 10–20% in most studies with more than 10 patients.^18,22,24,26,28–31^ One article, presenting the outcomes of 63 patients, had 100% survival rates and 100% primary healing rates.^27^ The different flaps for wound closure were not compared for the same surgical technique although the choice of flap, independent of the surgical technique, may also have impacted the healing rates.

All techniques had similar revision rates from TKA to AKA under 20%.^9,12,30,17,19,21,23–27^ A subset of articles report maintenance of gait measures. Albino et al. (2014) noted that trauma patients and those under the age of 50 years were more likely to walk following their amputation, highlighting the importance of aetiology and patient factors in influencing the return to walking following amputation.^29^ Return to walking varies considerably between and within surgical techniques, with the classical approach reporting 47% of 41 patients fitted with a prosthesis,^18^ or 100% in six patients.^16^ The Mazet technique reports that either 70% of patients^22^ or approximately half of patients^24^ were fitted with a suction socket. Return to walking rates vary most with articles presenting the Gritti-Stokes technique. Some report that 22% of their patients go on to walk independently^29^and others that 55% of patients are fitted with a prosthesis and are using it.^26^ 60% of patients are deemed to have a ‘useful limb’ that can easily be used for transfers and/or fitted with a prosthesis,^31^ while some report that as high as 82% of their patients were referred to a limb fitting centre for prosthetic prescriptions.

Pain is an important consideration for quality of life after an amputation,^36^ but we found no studies reporting pain, nerve management strategies or neuroma formation.

The mixed results from this review highlight the need for robust prospective studies with objective outcome measures to compare the different TKA surgical techniques for subsequent comparison to AKA. Each article presented has different age distributions and patient populations (dysvascular and/or trauma), limiting comparisons and conclusions. Surgical practice has changed and the early reports were from 1966 and the latest in 2019; over this time period, the practice of surgery has changed and seen accompanying improvements in key adjuncts and pathways, affecting outcomes and survival rates.

### Biomechanical outcomes

Biomechanical outcomes provide objective data of the functional levels of TKA but are sparse. Pinzur (1993) reported gait spatiotemporal parameters, including speed, stride length and cadence in patients with mid-foot, Syme’s, below-, through- and above-knee amputations. Their results indicated that these parameters were larger in more distal amputations. The TKA participants were all amputated following the classical approach and thus TKA surgical techniques were not compared.^37^ The same team found that the energy expenditure of TKA was midway between below-knee amputation (BKA) and AKA.^38^ A third study by Pinzur and colleagues found, surprisingly, that vascular insufficiency patients with TKA were more likely to maintain walking independence than those with BKA.^39^ However, a more recent study comparing four ex-military trauma TKA participants to matched AKA participants found no difference in gait velocity, cadence, stride length, stride width or work of ambulation.^40^ A study with 13 veterans with unilateral amputation found no correlation between residual femur length and temporal-spatial, kinematic or kinetic parameters.^41^ Finally, another study made a case against TKA, comparing 18 participants with TKA to 34 participants with AKA. TKA led to lower self-selected walking speeds and questionnaires found lower independence in transfers, walking and stair-climbing.^42^ TKA did however lead to lower reported pain levels. Published studies are again limited by number of participants and have only examined gait parameters including speed, stride length and cadence or energy expenditure.^37,38,40,43^ These heterogenous and contradictory findings again highlight the need for further investigations into the biomechanical outcomes of TKA.

## Discussion

Regardless of the technique performed, TKA affords theoretical biomechanical advantages, but these have not been confirmed through robust studies. TKA is postulated to facilitate weight bearing on the distal end of the femur; however, if return to walking is unlikely, then TKA also provides a longer lever arm, facilitating transfers and an improved seated posture with balance control. TKA is also a less traumatic amputation than AKA in that fewer musculoskeletal structures are surgically insulted. Rather, the hip muscles are preserved in TKA, likely allowing for improved hip muscle control and function compared to AKA.^5,44^ Hip flexion contractures, a common complication of AKA, are also less common with TKA.^14^

While these important advantages have implications for long-term functional outcomes, there has been no research to date to verify these theories. Further, TKA also presents disadvantages. For example, there can be challenges with prosthetic fitting, although advances in socket, prosthetic knee and prosthetic ankle design can mitigate this. With TKA, the prosthetic knee joint is not aligned with the contralateral healthy knee and the longer thigh can make it more difficult to stand from a sitting position while wearing a prosthetic limb. Preserving the femoral condyles also increases the volume of the residual limb, resulting in the need for a larger prosthetic socket. However, the Mazet and Gritti-Stokes surgical techniques claim to reduce volume of the residual limb by shortening the femur and reducing the bulge of the condyles by shaving or removing them completely, although the bulbous condyles can provide a better prosthetic suspension.^15^

Few outcome data relating to TKA are reported and coupled with the absence of a standardised surgical technique; comparison of TKA to AKA is not possible. The variations in TKA surgical technique (classical, Mazet or Gritti-stokes, with their own respective modifications) compound this challenge.

If walking is a goal following amputation, TKA may facilitate end load bearing. Should TKA fail or load bearing of the residual limb be too painful, the patient can still be fitted as an AKA with a longer residual limb, which is advantageous due to the longer lever arm.^19^ Distal weight-bearing on the femur also potentially improves bone health, consequently improving long-term health between AKA and BKA.^45^ However, further research is required to substantiate these ideas.

Osseointegration following amputation is an alternative to TKA or AKA but there is no comparison of outcomes between TKA and osseointegrated AKA. Osseointegration remains a growing topic of interest that also requires further study but it has its own inherent complications and limitations.^46^

To better define the place of TKA in the surgical decision making when performing MLLA, we recommend the following:

• Reporting of the outcomes of each surgical technique to understand when each technique is appropriate or should not be considered.
• Quantification of the biomechanics and health of people with TKA compared to people with AKA or BKA. These metrics are important in predicting long-term health outcomes, which is especially relevant to TKA as this procedure is often performed in younger patients than AKA.
• Comparisons of surgical, functional and biomechanical outcomes should be undertaken in patients with the same aetiology, age and physical level before amputation when possible. Young, otherwise healthy trauma patients may have different outcomes than older dysvascular patients with co-morbidities. However, the low number of TKAs performed makes matching of participant cohorts difficult. Thus, cross-sectional and longitudinal study designs should consider aetiology, age and physical level in results interpretation.
• Early discussions with the different actors in the rehabilitation process following MLLA have demonstrated a lack of consensus between surgeons, physiotherapists, prosthetists and patients. Often contradicting opinions are given for or against TKA with little objective data to support each opinion.

## Conclusion

Through-knee amputation is still performed and represents less than 5% of all major lower limb amputations. Different techniques exist for TKA, with the three main approaches presented in this review. Their influence on the clinical, functional and biomechanical outcomes is unknown and it remains unclear whether TKA leads to better outcomes than AKA. Further study of the influence of the surgical techniques on these outcomes is important. Currently, there is no clear evidence to suggest that TKA is a preferable amputation level when below-knee amputation is not feasible and there is no clear evidence to suggest it is preferable to an above-knee amputation. Thus, the future and potential of this amputation technique remains uncertain. To address this uncertainty, TKA surgical techniques need to be thoroughly documented when presenting outcomes and in comparing TKA to AKA. This information is critical to guide future surgical decision making in major lower limb amputation and improve the subsequent design of sockets and prostheses, with a view to optimising outcomes.

## Figures and Tables

**Figure A1.**
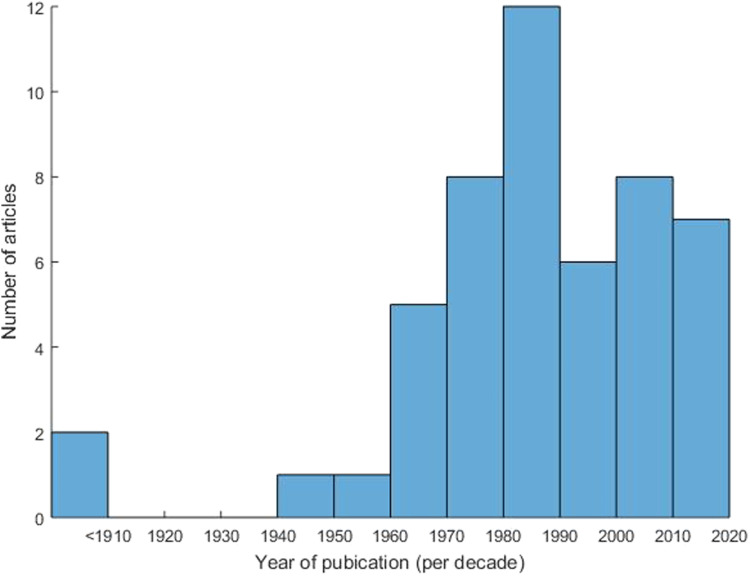
Number of articles presenting through-knee amputation (TKA) surgical techniques or modifications per decade. Articles not in English or French are included in this total (n = 10) but were not included in the subsequent review. Articles before 1940 were identified through reference screening of identified studies techniques to be included in the review.

## Appendix 1

1. (knee* adj3 amput*).mp. [mp=title, abstract, heading word, drug trade name, original title, device manufacturer, drug manufacturer, device trade name, keyword, floating subheading word, candidate term word]
2. (knee* adj3 disarticulat*).mp. [mp=title, abstract, heading word, drug trade name, original title, device manufacturer, drug manufacturer, device trade name, keyword, floating subheading word, candidate term word]
3. (knee* adj3 exarticulation*).mp. [mp=title, abstract, heading word, drug trade name, original title, device manufacturer, drug manufacturer, device trade name, keyword, floating subheading word, candidate term word]
4. (lower limb* adj3 amput*).mp. [mp=title, abstract, heading word, drug trade name, original title, device manufacturer, drug manufacturer, device trade name, keyword, floating subheading word, candidate term word]
5. (lower adj3 extremit* adj3 amput*).mp. [mp=title, abstract, heading word, drug trade name, original title, device manufacturer, drug manufacturer, device trade name, keyword, floating subheading word, candidate term word]
6. knee amputation/
7. TKAmp.mp.
8. 1 or 2 or 3 or 4 or 5 or 6 or 7
9. knee*.mp.
10. 8 and 9
11. surgery/
12. surg*.mp.
13. surgical technique/ or technique*.mp.
14. myocutaneous flap/ or myocutaneous.mp.
15. fasciocutaneous flap/ or myofasciocutaneous.mp.
16. operat*.mp.
17. sagittal*.mp.
18. mazet.mp.
19. gritti stokes.mp.
20. 11 or 12 or 13 or 14 or 15 or 16 or 17 or 18 or 19
21. 10 and 20

## Appendix 2

PRISMA flow diagram showing identification, screening, eligibility and inclusion of studies of through-knee amputation (TKA) surgical techniques, with reasons for exclusion.

## Appendix 3

Number of articles presenting through-knee amputation (TKA) surgical techniques or modifications per decade. Articles not in English or French are included in this total (*n* = 10) but were not included in the subsequent review. Articles before 1940 were identified through reference screening of identified studies. Articles prior to 1960 were not in English or French or did not present sufficient information on surgical techniques to be included in the review.
